# The risk of adverse pregnancy outcomes in women who are overweight or obese

**DOI:** 10.1186/1471-2393-10-56

**Published:** 2010-09-17

**Authors:** Chaturica Athukorala, Alice R Rumbold, Kristyn J Willson, Caroline A Crowther

**Affiliations:** 1 Discipline of Obstetrics and Gynaecology, The University of Adelaide, Adelaide, 5005, South Australia; 2 Menzies School of Health Research, PO Box 41096, Casuarina, Northern Territory, 0810, Australia; 3 Discipline of Public Health, The University of Adelaide, Adelaide, 5005, South Australia

## Abstract

**Background:**

The prevalence of obesity amongst women bearing children in Australia is rising and has important implications for obstetric care. The aim of this study was to assess the prevalence and impact of mothers being overweight and obese in early to mid-pregnancy on maternal, peripartum and neonatal outcomes.

**Methods:**

A secondary analysis was performed on data collected from nulliparous women with a singleton pregnancy enrolled in the Australian Collaborative Trial of Supplements with antioxidants Vitamin C and Vitamin E to pregnant women for the prevention of pre-eclampsia (ACTS). Women were categorized into three groups according to their body mass index (BMI): normal (BMI 18.5-24.9 kg/m^2^); overweight (BMI 25-29.9 kg/m^2^) and; obese (BMI 30-34.9 kg/m^2^). Obstetric and perinatal outcomes were compared by univariate and multivariate analyses.

**Results:**

Of the 1661 women included, 43% were overweight or obese. Obese women were at increased risk of pre-eclampsia (relative risk (RR) 2.99 [95% confidence intervals (CI) 1.88, 4.73], p < 0.0001) and gestational diabetes (RR 2.10 [95%CI 1.17, 3.79], p = 0.01) compared with women with a normal BMI. Obese and overweight women were more likely to be induced and require a caesarean section compared with women of normal BMI (induction - RR 1.33 [95%CI 1.13, 1.57], p = 0.001 and 1.78 [95%CI 1.51, 2.09], p < 0.0001, caesarean section - RR 1.42 [95%CI 1.18, 1.70], p = 0.0002 and 1.63 [95%CI 1.34, 1.99], p < 0.0001). Babies of women who were obese were more likely to be large for gestational age (LFGA) (RR 2.08 [95%CI 1.47, 2.93], p < 0.0001) and macrosomic (RR 4.54 [95%CI 2.01, 10.24], p = 0.0003) compared with those of women with a normal BMI.

**Conclusion:**

The rate of overweight and obesity is increasing amongst the Australian obstetric population. Women who are overweight and obese have an increased risk of adverse pregnancy outcomes. In particular, obese women are at increased risk of gestational diabetes, pregnancy induced hypertension and pre-eclampsia. Effective preventative strategies are urgently needed.

**Trial Registration:**

Current Controlled Trials ISRCTN00416244

## Background

In Australia, the incidence of overweight and obesity is increasing in both women and men and amongst all age groups [1]. Between 2004 and 2005, approximately 15% of women aged 25 to 34 years of age were obese [1]. This age bracket has the highest fertility rate and hence maternal overweight and obesity has significant implications for the provision of obstetric care [1].

Mothers who are overweight or obese during pregnancy and childbirth, as measured by increasing maternal body mass index (BMI), are known to be at risk of significant antenatal, intrapartum, postpartum and neonatal complications. Antenatal complications include recurrent miscarriage, congenital malformations, pregnancy induced hypertension (PIH), pre-eclampsia, gestational diabetes mellitus (GDM) and venous thromboembolism [2-5]. Overweight and obese women are more likely to be induced and require a caesarean [6,7]. Infants of overweight and obese mothers are often macrosomic and require prolonged hospital admissions [6,7].

It has been estimated that the cost of prenatal care in overweight women exceeds that of normal-weight control subjects by 5.4 to 16.2-fold depending on the degree of obesity [8]. In addition, children who are large for gestational age (LFGA) at birth, and hence likely to be exposed to an intrauterine environment of either diabetes or maternal obesity, are at increased risk of developing a metabolic syndrome, thus perpetuating the cycle of obesity and insulin resistance in subsequent generations [9].

The aim of this study was to assess the prevalence and impact of mothers being overweight and obese in early to mid-pregnancy on maternal, peripartum and neonatal outcomes in a sample of nulliparous women. An additional aim was to identify maternal characteristics associated with a high risk of being overweight and obese.

## Methods

1877 women were enrolled in the Australian Collaborative Trial of Supplements with antioxidants Vitamin C and Vitamin E to pregnant women for the prevention of pre-eclampsia (ACTS), a multi-centre randomised placebo controlled trial of antioxidant supplements for the prevention of perinatal complications [10]. The study recruited nulliparous women with a singleton pregnancy between 14 and 22 weeks gestation who were normotensive at the first measurement in pregnancy and again at trial entry. Of these women, 1661 (88%) had a BMI recorded at first antenatal visit and were included in the present study. Study recruitment took place between December 2001 and January 2005.

Women were randomised through a central telephone randomization service to either the vitamin (100 mg vitamin C and 400 mg vitamin E daily) or placebo group (four tablets of microcrystalline cellulose daily). The primary results of this trial have been published previously [10]. The trial found no overall difference in perinatal outcomes between antioxidant and vitamin groups, therefore data from both groups were combined in the analyses for the current study. Women were categorized into three groups according to their body mass index (BMI) which was calculated using hospital data from their first antenatal visit: normal (BMI 18.5-24.9 kg/m^2^); overweight (BMI 25-29.9 kg/m^2^) and; obese (BMI greater than 30 kg/m^2^).

### Data collection

Sociodemographic variables were collected either from women's medical records or self-completed questionnaires at trial entry and included: maternal age, ethnicity, body mass index, social-economic status as measured by Socio-Economic Indexes for Area (SEIFA) score [11], maternal education, smoking status and blood pressure at trial entry.

### Outcome variables

Pregnancy outcomes assessed included: maternal adverse outcome (a composite outcome defined as any of the following until six weeks postpartum: death, pulmonary oedema, eclampsia, stroke, thrombocytopenia, renal insufficiency, respiratory arrest, placental abruption, abnormal liver function, preterm prelabour rupture of membranes, major postpartum haemorrhage, postpartum pyrexia, pneumonia, deep-vein thrombosis, or pulmonary embolus requiring anticoagulant therapy); pregnancy induced hypertension (PIH); pre-eclampsia (defined as systolic blood pressure ≥ 140 mmHg or diastolic blood pressure [Korotkoff V] ≥ 90 mmHg on at least two occasions four or more hours apart, or both arising after 20 weeks' gestation and one or more of the following: proteinuria, renal insufficiency, liver disease, neurological problems, haematologic disturbances, or fetal growth restriction) [12]; antenatal hospitalisation; preterm prelabour rupture of the membranes; induction of labour; mode of birth; postnatal complications such as postpartum haemorrhage and infection; and length of hospital stay.

Neonatal outcomes included a composite outcome of death or infant adverse outcome defined as: fetal or perinatal death, birthweight < 3^rd ^centile for gestational age, severe respiratory distress syndrome, chronic lung disease, intraventricular hemorrhage grade 3 or 4, cystic periventricular leukomalacia, retinopathy of prematurity grade 3 or 4, necrotizing enterocolitis, 5 minute Apgar score < 4, seizures before 24 hours of age or requiring 2 or more drugs to control, hypotonia for ≥ 2 hours, stupor, decreased response to pain or coma, tube feeding for ≥ 4 days, care in the neonatal intensive care unit (NICU) > 4 days, or use of ventilation for ≥ 24 hours [13,14]; as well as: gestational age at birth; preterm birth (< 37 weeks);5 minute Apgar score < 7; infant body size at birth (weight, length and head circumference); small and large-for-gestational age (defined as a birth weight below the 10^th ^percentile or above 90^th ^percentile for gestation according to fetal sex on standardized birth-weight charts, respectively) [15]; macrosomia (defined as birthweight ≥ 4.5 kg); admission to NICU or neonatal nursery; respiratory distress syndrome; and length of hospital stay.

### Statistical analysis

Statistical analysis was carried out using SAS software, version 9.1. Binary variables were analysed using log-binomial regression with results expressed as relative risks and 95% confidence intervals. Continuous variables, if normally distributed, were analysed using analysis of variance and presented as risk-adjusted mean differences with 95% confidence intervals. Non-parametric tests were used for skewed data. A p value of < 0.05 was considered to indicate statistical significance. The group of women with a normal BMI was used as the reference category for all analyses.

## Results

### Demographics

Of the 1661 women included in the study, 943 (57%) had a normal BMI, 446 (27%) were overweight and 272 (16%) were obese at first antenatal visit (Table 1). No women recorded a BMI less than 18.5. The mean gestational age at trial entry was 17.2 weeks in all three groups.

**Table 1:** Demographics of women with normal BMI compared with overweight and obese women

*Characteristic*	*Normal* n = 943 (%)	*Overweight* n = 446 (%)	*Obese* n = 272 (%)	*Overweight* *vs. Normal* *[95% CI]*	*p value*	*Obese* *vs. Normal* *[95% CI]*	*p value*
Age (years)^a^	26.7 ± 5.9	26.8 ± 5.6	26.4 ± 5.1	0.2 [-0.5,0.8]	0.61	-0.3 [-1.1,0.5]	0.44
GA Entry (weeks)^a^	17.2 ± 2.4	17.2 ± 2.4	17.2 ± 2.5	-0.04 [-0.3,0.2]	0.77	-0.05 [-0.4,0.3]	0.77
Systolic BP at Trial Entry (mm Hg)^a^	108.2 ± 10.2	111.8 ± 9.9	116.0 ± 9.7	3.6 [2.5,4.8]	< 0.0001	7.8 [6.4,9.2]	< 0.0001
Diastolic BP at Trial Entry (mm Hg)^a^	63.8 ± 7.6	66.3 ± 7.9	70.0 ± 7.8	2.5 [1.6,3.4]	< 0.0001	6.1 [5.1,7.2]	< 0.0001
Race							
Caucasian	885 (93.8)	430 (96.4)	261 (96.0)	1.03 [1.00,1.05]	0.03	1.02 [0.99,1.05]	0.14
Asian	38 (4.0)	6 (1.3)	4 (1.5)	0.33 [0.14,0.78]	0.01	0.36 [0.13,1.01]	0.05
Other	20 (2.1)	10 (2.2)	7 (2.6)	1.06 [0.50,2.24]	0.88	1.21 [0.52,2.84]	0.66
Education							
Secondary or lower	394 (42.8)	182 (41.8)	124 (46.1)	0.98 [0.86,1.12]	0.74	1.08 [0.93,1.25]	0.33
Diploma or equiv	199 (21.6)	118 (27.1)	77 (28.6)	1.26 [1.03,1.53]	0.02	1.32 [1.06,1.66]	0.01
University	328 (35.6)	135 (31.0)	68 (25.3)	0.87 [0.74,1.03]	0.10	0.71 [0.57,0.89]	0.003
Socio-economic index							
Low SEI	222 (23.5)	112 (25.1)	96 (35.3)	1.07 [0.88,1.30]	0.52	1.50 [1.23,1.83]	0.0001
Low-Mid SEI	155 (16.4)	81 (18.2)	44 (16.2)	1.10 [0.87,1.41]	0.42	0.98 [0.72,1.34]	0.92
Mid-High SEI	248 (26.3)	119 (26.7)	71 (26.1)	1.01 [0.84,1.22]	0.88	0.99 [0.79,1.25]	0.95
High SEI	318 (33.7)	134 (30.0)	61 (22.4)	0.89 [0.75,1.05]	0.18	0.67 [0.52,0.84]	0.001
Smoking	198 (21.0)	94 (21.1)	51 (18.8)	1.00 [0.81,1.25]	0.97	0.89 [0.68,1.18]	0.42

^a^Value is mean ± standard deviation and the comparison is mean difference [95% CI].

BP, blood pressure; SEI, Socio-economic Index.

Overweight and obese women had significantly higher systolic blood pressure readings at trial entry compared with women with a normal BMI (Mean Difference (MD) 3.6 [95%CI 2.5,4.8], p < 0.0001 and MD 7.8 [95%CI 6.4,9.2], p < 0.0001 respectively). Similar findings were found for diastolic blood pressure readings at trial entry (MD 2.5 [95%CI 1.6,3.4], p < 0.0001 and MD 6.1 [95%CI 5.1,7.2], p < 0.0001 respectively) (Table 1).

Overweight women were more likely to be caucasian and less likely to be asian than normal weight women (RR 1.03 [95%CI 1.00, 1.05], p = 0.03 and RR 0.33 [95%CI 0.14, 0.78], p = 0.01 respectively). Obese women were less likely to be asian than women with a normal BMI (RR 0.36 [95%CI 0.13, 1.01], p = 0.05) (Table 1).

Overweight and obese women were more likely to have achieved a TAFE or equivalent education than healthy weight women (RR 1.26 [95%CI 1.03, 1.53], p = 0.02 and RR 1.32 [95%CI 1.06, 1.66], p = 0.01). Obese women were less likely to go to university than healthy weight women (RR 0.71 [95%CI 0.57,0.89], p = 0.003) (Table 1).

Obese women were more likely to be of low socio-economic level compared with normal BMI women (RR 1.50 [95%CI 1.23, 1.83], p = 0.0001). Accordingly, obese women were less likely to be of high Socio-Economic level compared with women with a normal BMI (RR 0.67 [95%CI 0.52,0.84], p = 0.001) (Table 1).

### Pregnancy outcomes

Obese women were at higher risk of developing pre-eclampsia compared with women with a normal BMI (RR 2.99 [95%CI 1.88, 4.73)], p < 0.0001). They were more likely to be hospitalised for hypertension than women with a normal BMI (RR 2.87 [95%CI 1.70, 4.84], p = 0.0001) (Table 2). Obese women received more magnesium sulphate and antihypertensives than women with a normal BMI (RR 2.97 [95%CI 1.01, 8.77], p = 0.05 and RR 3.31 [95%CI 1.85, 5.93], p = 0.0001 respectively) (Table 2).

**Table 2:** Pregnancy complications among women with normal BMI compared with overweight and obese women

*Characteristic*	*Normal* n = 943 (%)	*Overweight* n = 446 (%)	*Obese* n = 272 (%)	*Overweight* *vs. Normal* *[95% CI]*	*p value*	*Obese* *vs. Normal* *[95% CI]*	*p value*
Maternal Death or Adverse Outcome	68 (7.2)	43 (9.6)	28 (10.3)	1.34 [0.93,1.93]	0.12	1.43 [0.94,2.17]	0.10
Pre-Eclampsia	36 (3.8)	25 (5.6)	31 (11.4)	1.47 [0.89,2.42]	0.13	2.99 [1.88,4.73]	< 0.0001
Pregnancy Induced Hypertension	74 (7.8)	68 (15.2)	68 (25.0)	1.94 [1.43,2.65]	< 0.0001	3.19 [2.36,4.30]	< 0.0001
Severe PIH	13 (1.4)	17 (3.8)	15 (5.5)	2.76 [1.35,5.64]	0.01	4.00 [1.93,8.30]	0.0002
Antenatal hospitalisation hypertension	29 (3.1)	19 (4.3)	24 (8.8)	1.39 [0.79,2.44]	0.26	2.87 [1.70,4.84]	0.0001
MgSO4 used	7 (0.7)	4 (0.9)	6 (2.2)	1.21 [0.36,4.11]	0.76	2.97 [1.01,8.77]	0.05
2 hr OGTT ≥ 7.8 mmol/L	28 (3.0)	16 (3.6)	17 (6.3)	1.21 [0.66,2.21]	0.54	2.10 [1.17,3.79]	0.01
Antihypertensives	22 (2.3)	17 (3.8)	21 (7.7)	1.63 [0.88,3.05]	0.12	3.31 [1.85,5.93]	0.0001
PPROM	23 (2.4)	16 (3.6)	8 (2.9)	1.47 [0.78,2.76]	0.23	1.21 [0.55,2.67]	0.64

PIH, pregnancy induced hypertension; MgSO4, magnesium sulphate; OGTT, oral glucose tolerance test; PPROM, preterm prelabour rupture of membranes.

Compared to women with a normal BMI, overweight and obese women had an increased risk of pregnancy-induced hypertension (PIH) than women with a normal BMI (RR 1.94 [95%CI 1.43, 2.65], p < 0.0001 and RR 3.19 [95%CI 2.36, 4.30], p < 0.0001 respectively) and severe PIH (RR 2.76 [95%CI 1.35, 5.64], p = 0.01 and RR 4.00 [95%CI 1.93, 8.30], p = 0.0002 respectively) (Table 2).

Obese women were at higher risk of developing gestational diabetes than women with a normal BMI (RR 2.10 [95%CI 1.17, 3.79], p = 0.01) (Table 2).

Relating to induction of labour, overweight and obese women were more likely to be induced than women with a normal BMI (RR 1.33 [95%CI 1.13, 1.57], p = 0.001 and RR 1.78 [95%CI 1.51, 2.09], p < 0.0001 respectively) (Table 3). The indication was more commonly hypertension in overweight and obese women compared to women with a normal BMI (RR 1.93 [95%CI 1.21, 3.08], p = 0.01 and RR 3.96 [95%CI 2.57, 6.11], p < 0.0001 respectively). Overweight and obese women were more frequently induced for diabetes related complications than women with a normal BMI (RR 4.93 [95%CI 1.28, 18.99], p = 0.02 and RR 11.6 [95%CI 3.20, 41.69], p = 0.0002 respectively) (Table 3).

**Table 3:** Pregnancy complications among women with normal BMI compared with overweight and obese women

*Outcome*	*Normal* n = 943 (%)	*Overweight* n = 446 (%)	*Obese* n = 272 (%)	*Overweight* *vs. Normal* *[95% CI]*	*p value*	*Obese* *vs. Normal* *[95% CI]*	*p value*
Chorioamnionitis requiring antibiotics	10 (1.1)	3 (0.7)	3 (1.1)	0.63 [0.18,2.29]	0.49	1.04 [0.29,3.75]	0.95
Induction	250 (26.5)	157 (35.2)	128 (47.1)	1.33 [1.13,1.57]	0.001	1.78 [1.51,2.09]	< 0.0001
Induction for hypertension	35 (3.7)	32 (7.2)	40 (14.7)	1.93 [1.21,3.08]	0.01	3.96 [2.57,6.11]	< 0.0001
Induction for GDM/IDDM	3 (0.3)	7 (1.6)	10 (3.7)	4.93 [1.28,18.99]	0.02	11.6 [3.20,41.69]	0.0002
Caesarean Section	210 (22.3)	141 (31.6)	99 (36.4)	1.42 [1.18,1.70]	0.0002	1.63 [1.34,1.99]	< 0.0001
Elective Caesarean Section	51 (5.4)	30 (6.7)	18 (6.6)	1.24 [0.80,1.92]	0.33	1.22 [0.73,2.06]	0.45
Emergency Caesarean Section	159 (16.9)	111 (24.9)	81 (29.8)	1.48 [1.19,1.83]	0.0004	1.77 [1.40,2.23]	< 0.0001
CS for Pre-Eclampsia	9 (1.0)	3 (0.7)	9 (3.3)	0.70 [0.19,2.59]	0.60	3.47 [1.39,8.65]	0.01
CS for Fetal Distress	89 (9.4)	59 (13.2)	44 (16.2)	1.40 [1.03,1.91]	0.03	1.71 [1.23,2.40]	0.002
CS for Chorioamnionitis	2 (0.2)	0 (0.0)	1 (0.4)	--	--	--	--
CS for CPD	6 (0.6)	6 (1.3)	5 (1.8)	2.11 [0.69,6.52]	0.19	2.89 [0.89,9.39]	0.08
CS for Failure to Progress	83 (8.8)	59 (13.2)	44 (16.2)	1.50 [1.10,2.06]	0.01	1.84 [1.31,2.58]	0.0004
Length of Stay (days)^a^	3.0 ± 1.6	3.3 ± 2.1	3.4 ± 1.7	0.3 [0.1,0.5]	0.01	0.3 [0.1,0.6]	0.01
Major Post Partum Haemorrhage	20 (2.1)	12 (2.7)	10 (3.7)	1.27 [0.63,2.57]	0.51	1.73 [0.82,3.66]	0.15
Antibiotics post partum	132 (14.0)	81 (18.2)	48 (17.6)	1.30 [1.01,1.67]	0.04	1.26 [0.93,1.70]	0.13
Antibiotics for Wound Infection	10 (1.1)	6 (1.3)	8 (2.9)	1.27 [0.46,3.47]	0.64	2.77 [1.11,6.96]	0.03

^a^Value is mean ± standard deviation and the comparison is mean difference [95% CI].

GDM, gestational diabetes mellitus; IDDM, Insulin-Dependent Diabetes Mellitus; CS, caesarean section; IUGR, intrauterine growth restriction; CPD, cephalopelvic disproportion.

Compared with women with a normal BMI, overweight and obese women were more likely to undergo a caesarean section overall (RR 1.42 [95%CI 1.18, 1.70], p = 0.0002 and RR 1.63 [95%CI 1.34, 1.99] p < 0.0001 respectively) and have an emergency caesarean section (RR 1.48 [95%CI 1.19, 1.83], p = 0.0004 and 1.77 [95%CI 1.40, 2.23], p < 0.0001 respectively) (Table 3). Relating to the indication for caesarean section, obese women were more likely to require a caesarean section for pre-eclampsia than women with a normal BMI (RR 3.47 [95%CI 1.39, 8.65], p = 0.01), overweight and obese women were more likely to require a caesarean section for fetal distress than women with a normal BMI (RR 1.40 [95%CI 1.03, 1.91], p = 0.03 and RR 1.71 [95%CI 1.23, 2.40], p = 0.002) and overweight and obese women were more likely to require a caesarean section for failure to progress than women with a normal BMI (RR 1.50 [95%CI 1.10, 2.06], p = 0.01 and RR 1.84 [95%CI 1.31, 2.58], p = 0.0004 respectively) (Table 3).

Overweight women were more likely to require antibiotics postpartum compared to women with a normal BMI (RR 1.30 [95%CI 1.01, 1.67], p = 0.04) (Table 3). Obese women were more likely than women with a normal BMI to require antibiotics for a wound infection (RR 2.77 [95%CI 1.11, 6.96], p = 0.03) (Table 3).

Mean birth weight of babies born to overweight and obese mothers were significantly greater than babies born to women with a normal BMI (MD 64.4 ([95%CI -0.9, 129.8], p = 0.05 and MD 99.7 [95%CI 21.3, 178.2], p = 0.01 respectively) (Table 4). The birth weight Z-score of babies born to overweight and obese mothers was significantly greater than that of babies born to mother with a normal BMI (MD 0.10 [95% CI -0.00, 0.21], p = 0.06 and MD 0.24 [95% CI 0.11, 0.37], p = 0.0003 respectively) (Table 4). Babies of obese mothers were more likely to be large for gestational age (LFGA) compared with babies of women with a normal BMI (RR 2.08 [95%CI 1.47, 2.93], p < 0.0001) (Table 4). Babies of obese mothers were more likely to be macrosomic than those of mothers with a normal (RR 4.54 [95%CI 2.01, 10.24], p = 0.0003) (Table 4).

**Table 4:** Clinical outcomes among babies born to women with normal BMI compared to overweight and obese women

*Outcome*	*Normal* n = 943 (%)	*Overweight* n = 446 (%)	*Obese* n = 272 (%)	*Overweight* *vs. Normal* *[95% CI]*	*p value*	*Obese* *vs. Normal* *[95% CI]*	*p value*
Infant Death or Adverse Outcome	97 (10.3)	50 (11.2)	28 (10.3)	1.09 [0.79,1.50]	0.60	1.00 [0.67,1.49]	1.00
Death Pre-Discharge	12 (1.3)	6 (1.3)	8 (2.9)	1.06 [0.40,2.80]	0.91	2.31 [0.95,5.60]	0.06

Liveborns	n = 933 (%)	n = 441 (%)	n = 266 (%)				

GA at Delivery (weeks)^b^	40.0 (39.0-41.0)	40.1 (39.0-41.0)	40.0 (38.9-41.0)	--	0.34	--	0.72
Preterm birth (GA < 37 weeks)	59 (6.3)	27 (6.1)	21 (7.8)	0.97 [0.62,1.51]	0.90	1.24 [0.77,2.01]	0.37
Apgar at 5 minutes < 4	3 (0.3)	1 (0.2)	1 (0.4)	0.71 [0.07,6.76]	0.76	1.17 [0.12,11.19]	0.89
Admission to NICU	29 (3.1)	15 (3.4)	9 (3.4)	1.09 [0.59,2.02]	0.77	1.09 [0.52,2.27]	0.82
RDS	3 (0.3)	5 (1.1)	3 (1.1)	3.53 [0.85,14.69]	0.08	3.51 [0.71,17.28]	0.12
Birth Weight (g)^a^	3376 ± 573.7	3440 ± 553.5)	3476 ± 630.8	64.4 [-0.9,129.8]	0.05	99.7 [21.3,178.2]	0.01
SFGA (Birthweight < 10^th ^percentile)	91 (9.8)	35 (7.9)	20 (7.5)	0.81 [0.56,1.18]	0.28	0.77 [0.48,1.23]	0.27
LFGA (Birthweight ≥ 90^th ^percentile)	76 (8.1)	48 (10.9)	45 (16.9)	1.34 [0.95,1.88]	0.10	2.08 [1.47,2.93]	< 0.0001
Birthweight ≥ 4.5 kg	10 (1.1)	9 (2.0)	13 (4.8)	1.91 [0.78,4.67]	0.16	4.54 [2.01,10.24]	0.0003

Births (excluding fetal losses)	n = 940 (%)	n = 443 (%)	n = 269 (%)				

Birth Length (cm)^a^	50.4 ± 3.1	50.6 ± 3.0	50.5 ± 2.9	0.21 [-0.1,0.6]	0.24	0.17 [-0.3,0.6]	0.43
Birth Head Circumference (cm)^a^	34.4 ± 1.9	34.7 ± 1.8	34.7 ± 1.9	0.30 [0.1,0.5]	0.01	0.34 [0.1. 0.6]	0.01
Birth Weight Z-Score^a^	-0.08 ± 0.89	0.02 ± 0.94	0.16 ± 1.08	0.10 [-0.00,0.21]	0.06	0.24 [0.11,0.37]	0.0003
Birth Length Z-Score^a^	0.01 ± 1.18	0.08 ± 1.19	0.11 ± 1.16	0.08 [-0.06,0.21]	0.27	0.10 [-0.06,0.27]	0.21
Birth Head Circumference Z-Score^a^	-0.21 ± 1.10	-0.03 ± 1.13	0.07 ± 1.16	0.18 [0.05,0.31]	0.01	0.28 [0.13,0.44]	0.0003

^a^Value is mean ± standard deviation and the comparison is mean difference [95% CI].

^b^Value is median (Interquartile range).

SFGA, small for gestational age; NICU, neonatal intensive care unit; RDS, respiratory distress syndrome, GA, gestational age;

Babies of overweight and obese mothers had a greater head circumference when compared with babies of mothers with a normal BMI (MD 0.30 [95%CI 0.1, 0.5], p = 0.01 and MD 0.34 [95%CI 0.1, 0.6], p = 0.01 respectively) (Table 4). Accordingly, these finding were similar for birth head circumference Z-scores (MD 0.18 [95%CI 0.05, 0.31], p = 0.01 and MD 0.28 [95%CI 0.13, 0.44], p = 0.0003 respectively) (Table 4).

## Discussion

Our study found that increasing maternal BMI was associated with adverse health outcomes for both the mother and her baby. These findings are consistent with those of previous studies in showing an association between increasing maternal BMI and an increased risk of hypertensive disorders of pregnancy, GDM, induction of labour, caesarean section, longer length of maternal stay in hospital and increased birth weight [4-7]

The prevalence of maternal overweight and obesity found in this study (43%) is higher than that reported in one other study of Australian women giving birth in Queensland between 1998 and 2002, which reported that 34% of women were overweight or obese[7]. Recruitment for the ACTS trial took place between 2001 and 2005, therefore the higher prevalence seen in this study is consistent with national data indicating that overweight and obesity is increasing across the entire Australian population [1].

Our study found that Caucasian women were more likely to be overweight and obese than Asian women. The relationship of increasing BMI and its associated complications varies between ethnic groups. Asian populations have more fat and more comorbidities for any given BMI, resulting in different suggested BMI cut-off points for these populations [16]. We used the same BMI cut-off points for all races included in our study and hence Asian women are more likely to have been allocated to the normal BMI group.

We found that overweight and obese women were more likely to achieve a TAFE or equivalent education than healthy weight women and that obese women were less likely to obtain a university education than healthy weight women. Furthermore, obese women were more likely to be of low socio-economic level compared with women in the normal BMI group. This is in keeping with findings from a large, population-based study conducted in Australia that concluded that lower educational attainment was consistently predictive of obesity in each sex and that increasing income decreased the risk of obesity in women [17].

The findings of our study confirm the association between increasing BMI and the risk of PIH [4,6,7,18,19]. Despite detecting an increase in the risk of pre-eclampsia amongst obese women when compared with women with a normal weight, we were unable to detect a difference between the overweight and normal weight groups; however there did appear to be a trend towards increasing risk in the overweight group. A previous study has demonstrated a linear relationship between increasing BMI and pre-eclampsia amongst both overweight and obese women which is more pronounced amongst nulliparous women [19]. It is estimated that each one-unit increase in BMI among nulliparous women confers a 7% increase in risk for pre-eclampsia (95%CI 1.06, 1.08) and a 6% increase in risk for early pre-eclampsia (95%CI 1.05, 1.08) (13).

Our study found that obese women were at increased risk of developing gestational diabetes compared with women with a normal weight. We did not detect a difference in risk between the overweight and normal weight groups. A recent meta-analysis exploring the association between GDM and BMI estimated that the risk of developing GDM is two and four times higher among overweight and obese women respectively compared with normal-weight pregnant women [20]. Although our results showed an increased incidence of pre-eclampsia and GDM among obese women compared to normal weight women, it is likely that we were unable to detect the less pronounced variation in incidence between overweight women and their healthy counterparts due to inadequate statistical power resulting from small numbers of women in the overweight and obese groups.

The increased incidence of antenatal medical complications seen in overweight and obese mothers in our study contributed to increased rates of induction compared with women with a normal BMI. Overweight and obese mothers had higher rates of emergency caesarean for indications including pre-eclampsia, fetal distress and failure to progress. We did not see an increase in caesarean section for cephalo-pelvic disproportion (CPD) among overweight and obese mothers, as has been reported in previous research [21]. CPD in overweight and obese patients has been described as a consequence of increased fetal size and soft-tissue dystocia as a result of adipose tissue accumulation in the maternal pelvis [22].

Postpartum, women who are overweight and obese are more likely to suffer from infective complications and haemorrhage than women with a healthy weight [23-26]. In keeping with this, we found that women who were overweight were more likely to receive antibiotics postpartum compared with women with a normal weight and that obese women were more likely to receive antibiotics for wound infection compared with normal weight women. The increase in PPH seen with increasing BMI in other studies is thought to be contributed to by the higher rates of caesarean section and hence higher risk of haemorrhage [26]. Although increased haemorrhage rates amongst obese nulliparous women have been described [26], our study, which included only nulliparous women, did not find differences in risk of PPH.

There were no cases of maternal death in our sample, which was expected given our sample size and the low maternal mortality rate seen in the Australian obstetric population. The most recent report on maternal deaths in Australia between 1997 and 1999 found obstetric haemorrhage to be the leading direct cause of maternal mortality which was postulated to be a result of increasing caesarean rates [27]. Thromboembolism and hypertensive disorders were also identified as major contributors. Increasing BMI is associated with an increased risk of all three of these factors and therefore it is possible that with increasing obesity amongst pregnant women, a trend towards increasing maternal mortality may emerge. As yet, in Australia, state-based routine perinatal data collections do not include maternal BMI thus inhibiting an accurate assessment of the impact of weight on maternal death.

Consistent with the established association between increasing maternal BMI and increasing birth weight, our results showed a correlation with increasing head circumference [6,7]. Increased rates of vaginal birth among normal weight women may have contributed to this result as moulding of the baby's skull during vaginal delivery would have resulted in a smaller head circumference. Specific anthropomorphic patterns in offspring of diabetic mothers have been observed [28] however other measures of body size and skin fold thickness were not measured in this study. We did not collect data on rates of shoulder dystocia but increased rates amongst obese women as a result of soft-tissue dystocia and fetal macrosomia have been reported [24].

Although the sample size for this study was too small to assess the effect of increasing BMI on rare outcomes, we used a composite endpoint for maternal and infant serious outcome that included morbidities such as rates of venous thromboembolism, chorioamnionitis, NICU admission, neonatal death, stillbirth and infant death. Our study did not find a statistically significant difference in this composite endpoint, however increased rates of mortality and morbidity among overweight and obese mothers have been demonstrated by previous studies [5,7,29,30]. Our study did not see a correlation between increasing BMI and preterm birth which has previously been identified and thought to be a result of higher rates of early induction and preterm prelabour rupture of membranes (PPROM) [30].

## Conclusions

The results of our study add to the emerging body of literature on the consequences of mothers being overweight and obese during pregnancy and childbirth. Increased maternal and neonatal morbidity results in the increased utilisation of resources at a significant cost to the community. There is an urgent need to establish effective preventative strategies, both prior to pregnancy and during pregnancy, based on evidence from high quality randomised controlled trials.

## Competing interests

Dr Alice Rumbold and Professor Caroline Crowther were key investigators in the Australian Collaborative Trial of Supplements with antioxidants Vitamin C and Vitamin E to pregnant women for the prevention of pre-eclampsia (ACTS)

## Authors' contributions

CA and ARR contributed to the design of the study and drafted the manuscript. CAC conceived the idea of the study, participated in its design and coordination and helped to draft the manuscript. KJW performed the statistical analysis. All four authors read and approved the final manuscript.

## Pre-publication history

The pre-publication history for this paper can be accessed here:

http://www.biomedcentral.com/1471-2393/10/56/prepub
